# Fibrinogen-like protein 2 gene silencing inhibits cardiomyocytes apoptosis, improves heart function of streptozotocin-induced diabetes rats and the molecular mechanism involved

**DOI:** 10.1042/BSR20150078

**Published:** 2015-06-22

**Authors:** Zheng Zhenzhong, Yu Yafa, Liang Jin

**Affiliations:** * Department of Cardiology, the First Affiliated Hospital of Nanchang University, Nanchang, Jiangxi 330006, China; † The First Affiliated Hospital of Nanchang University, Nanchang, Jiangxi 330006, China; ‡ Department of Cardiology, the First People's Hospital of Fuzhou City, Fuzhou, Jiangxi 344000, China

**Keywords:** apoptosis, diabetes, fibrinogen-like protein 2, heart function, tumour necrosis factor ±, molecular mechanism

## Abstract

Fibrinogen-like protein 2 (Fgl2) is involved in apoptosis, angiogenesis and inflammatory response. Diabetes is closely associated with apoptosis, angiogenesis and coagulation. So it allowed us to assume that Fgl2 plays an important role during the process of diabetic cardiomyopathy (DCM). In the present study, we test that the feasibility of Fgl2 as a therapeutic target for the treatment of DCM and its possible molecular mechanism involved. We found that Fgl2 gene silencing inhibits apoptosis and improves heart function of streptozotocin (STZ)-induced diabetes rats, the possible mechanism maybe that Fgl2 gene silencing reduces the tumour necrosis factor (TNF)±levels, decreases the expression of B-cell lymphoma-2 (bcl2), bcl-2-associated X (bax), toll-like receptors 4 (TLR4) and p38 mitogen-activated protein kinase (MAPK). In conclusion, Fgl2 is a potent target to treat DCM.

## Abstract (precis)

We assumed that Fibrinogen-like protein 2 (Fgl2) plays an important role during the process of diabetic cardiomyopathy (DCM). In the present study, we test that the feasibility of Fgl2 as a therapeutic target for the treatment of DCM and its possible molecular mechanism involved.

## INTRODUCTION

Diabetic cardiomyopathy (DCM) is a complication of diabetes that affects heart function and contributes to mortality. Although both heart disease and diabetes are widely studied, how DCM develops is not well understood. Cardiomyopathy, a known symptom of diabetes, occurs when the muscles of the heart weaken and the heart is no longer strong enough to pump blood and properly circulate it throughout the body. Adults with diabetes are 2–4 times more likely to die of heart failure than the rest of the population. So it is very important to search for therapeutic targets for the prevention of diabetes.

Our study found that fibrinogen-like protein 2 (Fgl2) gene silencing activates angiopoietin/Tie system and induces myocardial microvascular endothelial Cells proliferation and cell migration, we also found that serum Fgl2 levels elevated in patients with acute coronary syndrome (ACS), these results are preliminary, but very meaningful [1,2]. Researchers discovered that excessive induction of Fgl2 under certain medical conditions (e.g., pathogen invasion) could trigger complement activation, inflammatory response, cellular apoptosis and immune dysfunctions. Fgl2 prothrombinase is a fibrinogen-like protein associated with lethality in hepatitis, spontaneous abortion (fetal loss syndrome) and graft rejection [3–5]. Prothrombinase activity is only demonstrable when the protein is associated with phospholipids, typified by cell membranes, coagulation factor V is required for maximal activity and this activity is independent of factor Xa [6]. Cells expressing Fgl2 prothrombinase include macrophages, vascular endothelial cells, fetal trophoblast and a subset of decidual stromal cells [3–5,7]. In conclusion, Fgl2 is involved in apoptosis, angiogenesis and inflammatory response. As we know, diabetes is closely associated with apoptosis, angiogenesis and coagulation [8–10]. So it allowed us to assume that Fgl2 plays an important role during the process of DCM.

In the present study, the purpose of the present study was to test that the feasibility of Fgl2 as a therapeutic target for the treatment of DCM and its possible molecular mechanism involved.

## MATERIALS AND METHODS

### Animals (streptozotocin-induced diabetes)

The present study is an experimental study and has been approved by the First Affiliated Hospital of Nanchang University Research and Ethical Committee. All experimental rats were kept in a temperature-controlled, air-conditioned conventional animal house with a 12-h light-dark cycle and given free access to food and water. Procedures were performed according to the recommendations of the institutional animal care committee. Thirty-two male Sprague–Dawley rats weighing 200–220 g were fasted for 10 h, lightly anaesthetized [ketamine 40 mg/kg intramuscularly (IM)] and injected intraperitoneally with freshly prepared streptozotocin (STZ) (Sigma Chemical Co; 65 mg/kg, *n*=8) or vehicle (0.1 mol/l citrate-phosphate buffer, pH 4.5, *n*=8) according to the references [11]. Blood glucose levels were monitored 72 h later after STZ or vehicle injection, at regular intervals throughout the study and immediately prior to killing. Blood samples were obtained by tail prick and blood glucose concentration was measured using a Changsha Sannuo stable blood glucose meter. Only those STZ-induced diabetic rats with serum glucose levels (≥16.7 mol/l) were included in the diabetic group (*n*=29), 24 of the 29 STZ-induced diabetic rats were randomly divided into diabetic group (DM group), Fgl2 gene silencing mediated by lentiviral vector group (Fgl2–RNAi-DM group), empty vector group (GFP-DM group).

### Materials

The Bax mouse monoclonal antibody, Bcl-2 mouse monoclonal antibody, Fgl2 mouse monoclonal antibody, Na^+^/K^+^-ATPase α rabbit polyclonal antibody and β-actin were purchased from Santa Cruz Biotechnology. Rabbit anti-TLR4, p38 MAPK rabbit polyclonal antibody was purchased from Beijing Zhongsha Jinqiao Company. Tumour necrosis factor (TNF)-α ELISA was purchased from Shanghai Senxiong Technology Company. Apoptosis of TUNEL cell detection kit was purchased from Promega. STZ was from Sigma; pGCSIL–Fgl2, pGC–LV (left ventricular), pHelper 1.0 and pHelper are provided by Jikai Gene Company.

### RNAi design and preparation and transfection of siRNAs

Fgl2 RNAi or GFP virus was provided by Jikai Company. Briefly, the nucleotide sequences of Fgl2 (NM_053455) were found in the GenBank. Target sequence of Fgl2 gene (Forward: 5′- ccggacTGGAGAGTCAGGTGAACAActcgagTTGTTCACCTG ACTCTCCAgttttttg -3′, Reverse: 5′-aattcaaaaaacTGGAGAGTCAGGTGAACAA ctcgagTTGTTCACCTGACTCTCCAgt-3′) could be effectively silenced with RNA interference were confirmed. The cDNA containing both sense and antisense Oligo DNA fragments of target sequences were designed and cloned into the pGCSIL–GFP vector which was digested by AgeI/EcoRI. The obtained lentiviral vector containing Fgl2–shRNA was confirmed by digestion and sequencing. Using lentiviral vector pHelper 1.0, pHelper2.0, pGC—LV–Fgl2 to transfect 293T cells, then the lentivirus were collected. The titre of virus was tested according to the expression level of GFP. Titres of the virus stocks were routinely1 × 10^9^ transduction units (TU)·ml^−1^.

### Echocardiography

Echocardiography was performed to evaluate cardiac structure and function in all animals involved in the study. The rats were weighed and anaesthetized with 10% chloral hydrate at 0.3 ml/100 g body weight. 2D and M-mode echocardiographic measurements were carried out with a commercially available 12 MHz linear array transducer *in vivo* imaging system (GEvivid7). The transthoracic echocardiography images were obtained via long- and short-axis views using standard echocardiography techniques [12]. Cardiac structure was principally evaluated by the left ventricular end diastolic and systolic diameters. The left ventricular systolic function was assessed according to ejection fraction (EF) and fractional shortening (FS) and the left ventricular diastolic function was determined by Doppler waveforms of mitral inflows, which were obtained from an apical four-chamber. Parameters included left ventricular end-diastolic and end-systolic dimensions (LVEDD and LVESD), left ventricular EF (LVEF) and FS (LVFS) and heart rate (HR).

### Blood sample and tissue processing

After echocardiography, the rats were anaesthetized by using pentobarbital. Then the chest was opened for collecting blood and heart tissue. The blood was centrifuged at 3000 ***g*** at 4°C for 15 min and the serum was obtained and stored at −70°C until ready for analysis. The heart tissue was quickly isolated and part was infused into 4% paraformaldehyde, whereas part was stored at −70°C quickly. After 48 h of infusion of 4% paraformaldehyde, the tissues were paraffin-embedded. Myocardial tissue sections were used for histological analysis.

### ELISA for TNF-α

TNF-α level in the samples were estimated with ELISA Kit for TNF-α (Shanghai Senxiong Technology company). Briefly, standards and samples were bound by the immobilized antibody and an enzyme-linked polyclonal antibody specific for the cytokine was added to the wells followed by a substrate solution yielding a coloured product. The intensity of the colour was measured at 450 nm. The sample levels were calculated from a standard curve and were corrected for protein concentration.

### HE (hematoxylin and eosin) staining

The condyle and articular disk were fixed in 10% buffered paraformaldehyde, decalcified with 10% EDTA at 4°C for 4 weeks and embedded in paraffin wax. The serial 5-μm sections were cut along the sagittal plane and stained with HE. The central portions of each stained section were examined under a light microscope.

### Immunohistochemistry

The samples were processed according to standardized protocols in the routine histopathology laboratory. Briefly, after formalin fixation, they were cut into 2-mm slices and embedded in paraffin. Each 5-μm thin slice was mounted on glass slides, deparaffinized in xylene and rehydrated by sequential rinses in absolute, 95%, 80% and 70% ethanol. Endogenous peroxidase activity was exhausted by incubation with 1% H_2_O_2_. Sections were then washed in PBS, pre-incubated in 1% BSA for 1 h and then incubated overnight on the shaker at room temperature with a polyclonal primary antibody (1:50 dilution) directed against Fgl2. The sections were then washed and incubated in biotinylated anti-rabbit or anti-goat secondary antibody (1:1500 dilution) for 2.5 h. The signal was amplified with an acidin-biotin-horseradish peroxidase procedure (vector) and visualized with diaminobenzidine as the chromogen. Negative control slides were included in all experiments in which test antibody was omitted and replaced by control irrelevant diluent. The immunostaining of Fgl2 were performed by grey scale test with a computer-assisted image analysis system (HMIAS-2000, Champion Medical Imaging Co).

### TUNEL staining

We carried out terminal deoxynucleotidyl transfer-mediated dUTP nick end-labelling (TUNEL) by use of *in situ* Cell Death Detection Kit (Promega), according to the manufacturer's instructions. Briefly, after deparaffinization and rehydration, the sections were treated with protease K at a concentration of 20 μg/ml for 15 min. The slides were immersed in TUNEL reaction mixture for 60 min at 37°C in a humidified atmosphere in the dark. Then DAPI was used to incubate the slides for 5 min to show the characteristic blue nuclear staining. The slides were analysed by fluorescence microscope. Then the obtained images were merged and analysed using ImageJ software. To evaluate the apoptosis index of heart TUNEL stained tissues, 10 random heart fields per tissue section were captured at the 400× magnification.

### Western blot analysis

Tissue samples from the heart of 3–5 rats per group were dissected. The tissues were homogenized in 500 μl of 25 mmol/l Tris/HCl, pH 7.4, containing 1% Triton X-100, 0.1% SDS, 2 mmol/l EDTA and 1% protease inhibitor cocktail (Sigma) and centrifuged at 14000 ***g*** for 30 min at 4°C. The supernatants were used for Western blot analysis, with specific antibodies detecting p38 MAPK, TLR4, Fgl2, bax, bcl-2. According to standard protocols, the blots were probed with various primary antibodies and the HRP-conjugated secondary antibodies were used to reveal the specific protein bands with ECL detection reagents. The intensities of the various protein bands were quantified by densitometry.

### Statistical analysis

Data were analysed using SPSS software (version 17; SPSS). All values were expressed as means±S.E.M. Statistical analysis was determined using Student's *t*test (when two groups were considered) or by one-way ANOVA followed by multiple comparisons with the Fisher's Least Significant Difference (LSD) post-hoc test. *P*<0.05 was considered significant.

## RESULTS

### STZ-induced diabetic rats

Among 32 rats treated with STZ, 29 rats developed hyperglycaemia, hyperuresis, weight loss and elevated levels of food and water intake compared with control. Their serum glucose levels are shown in Table 1. Blood glucose levels were significantly higher in all diabetic rats compared with the control rats, but were not significantly different among the diabetic groups (*P* > 0.05).

**Table 1:** The rats serum fasting blood glucose (mmol/l) Four, 8 and 12 weeks after Fgl2–RNAi gene silencing (W4, W8, W12), diabetes group (DM), diabetic empty vector group (DM-GFP), Fgl2–RNAi gene silencing group (DM–Fgl2–RNAi). Data represent means±S.E.M. ^#^*P*<0. 05 compared with normal; *P<0.05 compared with W4, W8; ^☆^*P* > 0.05 compared with DM group.

	DM (*n*=8)	DM-GFP (*n*=8)	DM–Fgl2–RNAi (*n*=8)	Normal (*n*=8)
W4	27.72±8.58	31.00±4.14	28.80±10.33	5.70±0.91
W8	28.22±4.97	27.45±8.17.	25.20±11.85	5.50±1.01
W12	15.75±1.85^#^*	19.80±5.35^#^*	20.80±10.89^#^*^☆^	5.85±0.88^＊^

### Echocardiography

With regard to EF and FS, both showed a trend toward gradual decline in the diabetic rats over the 12 weeks, with significant reductions (*P*< 0.05) after diabetes was induced and significant increase(*P*< 0.05) in Fgl2–RNAi gene silencing group compared with diabetes group (DM), diabetic empty vector group (DM-GFP; Table 2). HR decreased significantly and heart weight/body weight increased significantly (*P*< 0.05) in diabetes group (DM), diabetic empty vector group (DM-GFP) compared with normal control group. In addition, EF and FS increased significantly, heart weight/body weight decreased significantly (*P*< 0.05) in Fgl2–RNAi gene silencing group compared with diabetes group (DM), diabetic empty vector group (DM-GFP) (Table 2). In conclusion, Fgl2–RNAi gene silencing inhibits ventricular hypertrophy and remodelling, improve the heart function in diabetic rats.

**Table 2:** Haemodynamic variables and physiological parameters Data represent means±S.E.M. #*P*<0.05 compared with normal; *P<0.05 compared with DM–Fgl2–RNAi,^☆^*P* > 0.05 compared with GFP group, **^▽^***P*<0.001 compared with normal; **^＊^***P* > 0.05 compared with normal. Abbreviations: HR, heart rate; BW, body weight; bpm, beat per minute; SF, left ventricular fractional shortening.

Group	EF%	SF%	HR (bpm)	HW/BW (mg/g)
Normal (*n*=8)	81.99±1.763	45.56±3.351	294.33±16.99	2.668±0.949
DM–Fgl2–RNAi (*n*=8)	75.83±4.708^#^	39.50±3.834^#^	245.00±28.80*	3.157±0.799^#^
DM-GFP (*n*=8)	67.50±2.509^#^*	31.20±4.543^#^*	206.66±24.22**^▽^**^#^	3.813±0.169^#^*
DM (*n*=8)	66.16±2.316^#^*^☆^	33.50±0.836^#^*^☆^	211.66±31.88^▽^*^☆^	3.536±0.535^#^*

### ELISA for TNF-α

TNF-α levels in diabetic group and GFP empty vector groups were significantly higher than normal (*P*<0.05) and the TNF-α levels were significantly higher in Fgl2 gene silencing group compared with normal group (*P*<0.05), but lower when compared with diabetic group and GFP empty vector group (*P*<0.05; Figure 1). These data indicated that Fgl2 gene silencing reduce the TNF-α levels.

**Figure 1:**
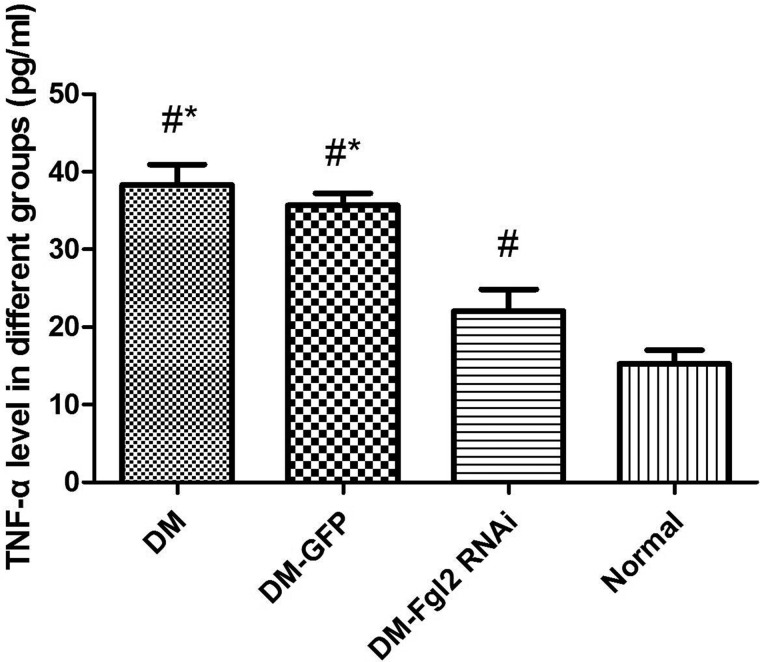
ELISA for TNF-α Data represent means±S.D. #*P*<0.05 compared with normal; *P<0.05 compared with DM–Fgl2–RNAi,^☆^*P* > 0.05 compared with GFP group, ^▽^*P*<0.001 compared with normal; ^＊^*P* > 0.05 compared with normal

### Fgl2 expression in heart tissues assessed by immunohistochemistry

Fgl2 expression in the heart was assessed by immunohistochemistry. Paraffin sections were reviewed and collected from the rats to evaluate Fgl2 expression in heart tissues. Results of immunohistochemistry demonstrated that Fgl2 was primarily localized in infiltrating interstitial and endothelial cells of the microvasculature (Figure 2). The immunostaining of Fgl2 were performed by grey scale test with a computer-assisted image analysis system and the results showed that the levels of Fgl2 were significantly (*P*<0.01) up-regulated in the diabetic rats compared with Fgl2–RNAi gene silencing group (178.20±2.28) and the normal controls (182.20±5.26), whereas there was no significant difference (*P* > 0.05) between those of the diabetic rats group (157.60±17.52) and diabetic empty vector group (DM-GFP; 159.80±2.77; *P* > 0.05). These data indicated that Fgl2–RNAi gene silencing decreased the expression of Fgl2.

**Figure 2:**
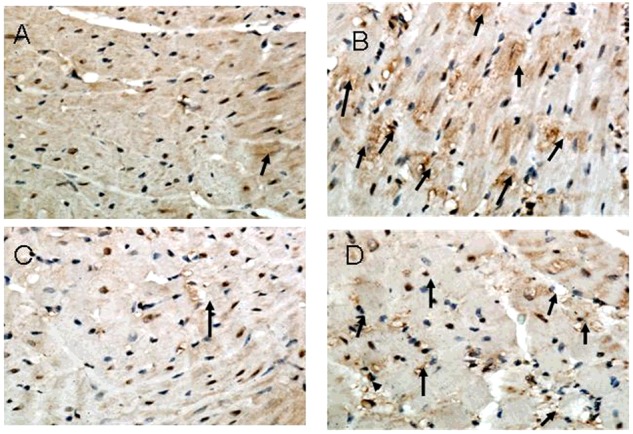
Fgl2 expression in the heart tissues in different groups, identified by immunochemical staining (×400) Arrows indicate Fgl2-positive cells. (**A**) Normal control. (**B**) DM-GFP group. (**C**) DM–Fgl2–RNAi group. (**D**) DM group.

### HE staining and TUNEL staining

HE staining showed that cell morphology is normal in normal group, whereas in diabetic rats groups myocardial cells disorder, cardiac hypertrophy are seen, monocyte infiltration is observed are also seen, at the same time we can see that monocyte infiltration decreased significantly in Fgl2–RNAi gene silencing group (Figure 3).

**Figure 3:**
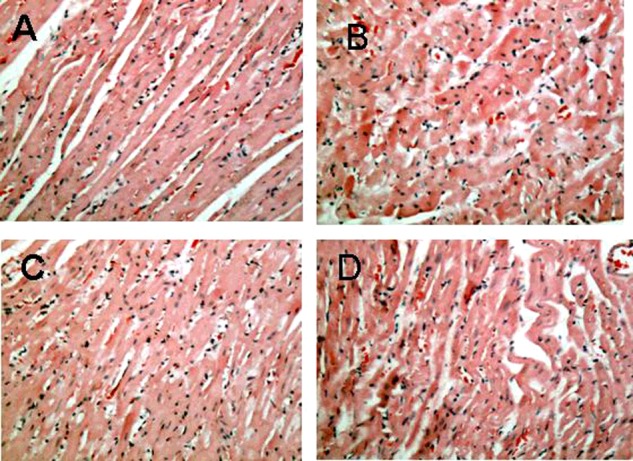
HE staining in different groups Results showed that cell morphology is normal in normal group, whereas in diabetic rats groups, myocardial cell disorder, cardiac hypertrophy are seen and monocyte infiltration is observed; at the same time we can see that monocyte infiltration decreased significantly in Fgl2–RNAi gene silencing group. (**A**) Normal control. (**B**) DM-GFP group. (**C**) DM–Fgl2–RNAi group. (**D**) DM group.

TUNEL examination was carried out to examine the apoptosis of cardiomyocytes. TUNEL staining results showed that few TUNEL-positive cells could be observed in the normal group. Importantly, the TUNEL-positive cells significantly increased in diabetic group and GFP empty vector group compared with normal group (Figures 4A–4D). The TUNEL-positive cells decreased in the Fgl2 gene silencing compared with the diabetic group and GFP empty vector group (Figure 4E). This shows that Fgl2 gene silencing inhibits apoptosis.

**Figure 4:**
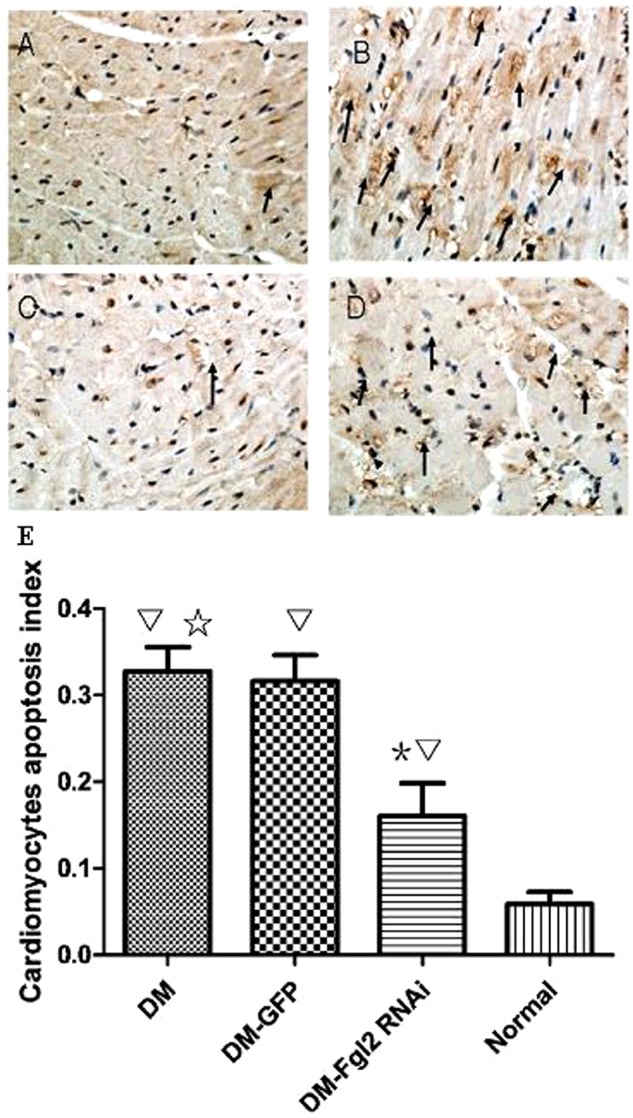
Cardiomyocytes apoptosis index in different groups (**A**) Normal control. (**B**) DM–Fgl2–RNAi group. (**C**) DM-GFP group. (**D**) DM group. TUNEL-positive cells were increased in in DM-GFP group and DM group. (**A**–**D**) Original magnification ×400; graphical representation of relative number of TUNEL-positive cells. Data are given as the means±S.D.s (**E**). ^#^*P*<0.05 compared with normal; *P<0.05 compared with DM–Fgl2–RNAi,^☆^*P* > 0.05 compared with GFP group, ^▽^*P*<0.001 compared with normal; ^＊^*P* > 0.05 compared with normal.

### The expression of Fgl2 and protein and apoptosis-related proteins

The expression of Fgl2 decreased significantly in DM–Fgl2–RNAi group compared with GFP group and DM group (*P*<0.05; Figure 5). The expression of B-cell lymphoma-2 (bcl2) decreased significantly in DM–Fgl2–RNAi group compared with GFP group and DM group (*P*<0.05; Figure 5). The expression of bax decreased significantly in DM–Fgl2–RNAi group compared with GFP group and DM group (*P*<0.001; Figure 5). We also found that the expression of bcl-2-associated X (bax) decreased significantly more than the expression of bcl2. These data indicate that Fgl2 gene silencing increases the ratio of bcl2:bax.

**Figure 5:**
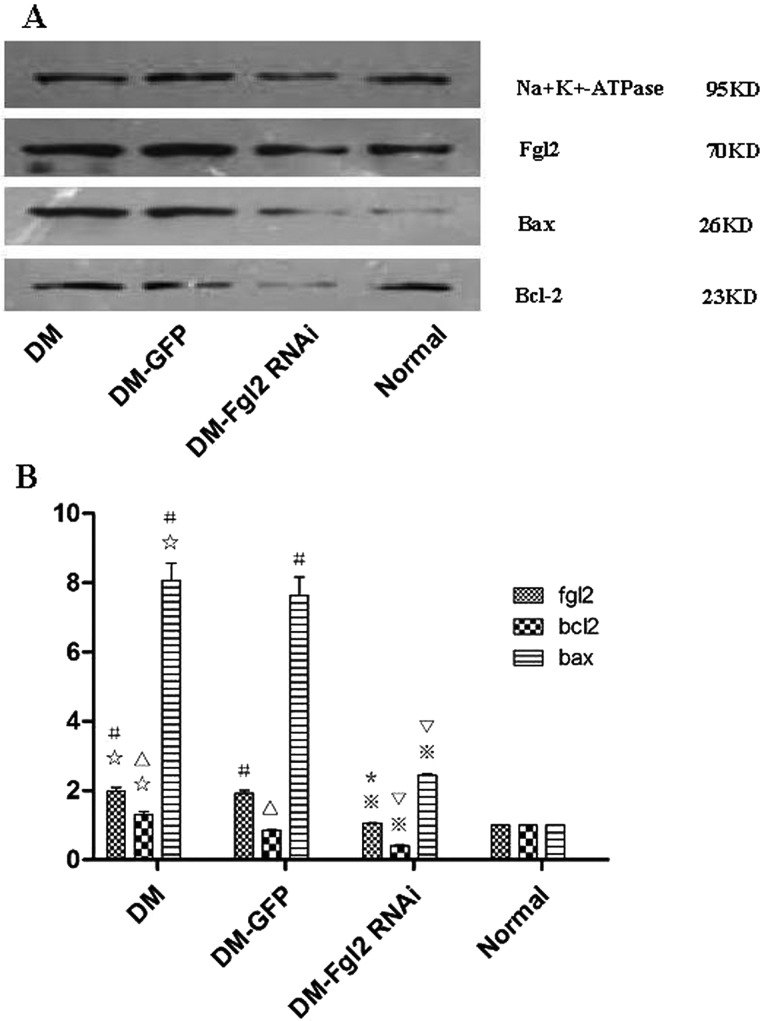
Expression of Fgl2 protein and apoptosis-related proteins Na^+^/K^+^-ATPase is used as a standard control. The expression of Fgl2 decreased significantly in DM–Fgl2–RNAi group compared with GFP group and DM group (*P*<0.05). The expression of bcl2 decreased significantly in DM–Fgl2–RNAi group compared with GFP group and DM group (*P*<0.05). The expression of bax decreased significantly in DM–Fgl2–RNAi group compared with GFP group and DM group (*P*<0.001). We also found that the expression of bax decreased significantly more than the expression of bcl2. Data are given as the means±S.D.s (**E**). #*P*<0.05 compared with normal; *P<0.05 compared with DM–Fgl2–RNAi, ^☆^*P* > 0.05 compared with GFP group, ^▽^*P*<0.001 compared with normal; ^＊^*P* > 0.05 compared with normal.

### The expression of TLR4 and p38 MAPK protein

In addition, we found that the expression of TLR4 and p38 MAPK protein increased significantly in GFP group and DM group compared with normal control group (*P*<0.05). The expression of toll-like receptors 4 (TLR4) decreased significantly in DM–Fgl2–RNAi group compared with GFP group and DM group (*P*<0.05; Figure 6). The expression of p38 MAPK (mitogen-activated protein kinase) in DM–Fgl2–RNAi group decreased significantly compared with GFP group and DM group (*P*<0.05; Figure 6). These data showed that TLR4 and p38 MAPK are involved in the process which Fgl2 gene silencing trigger.

**Figure 6:**
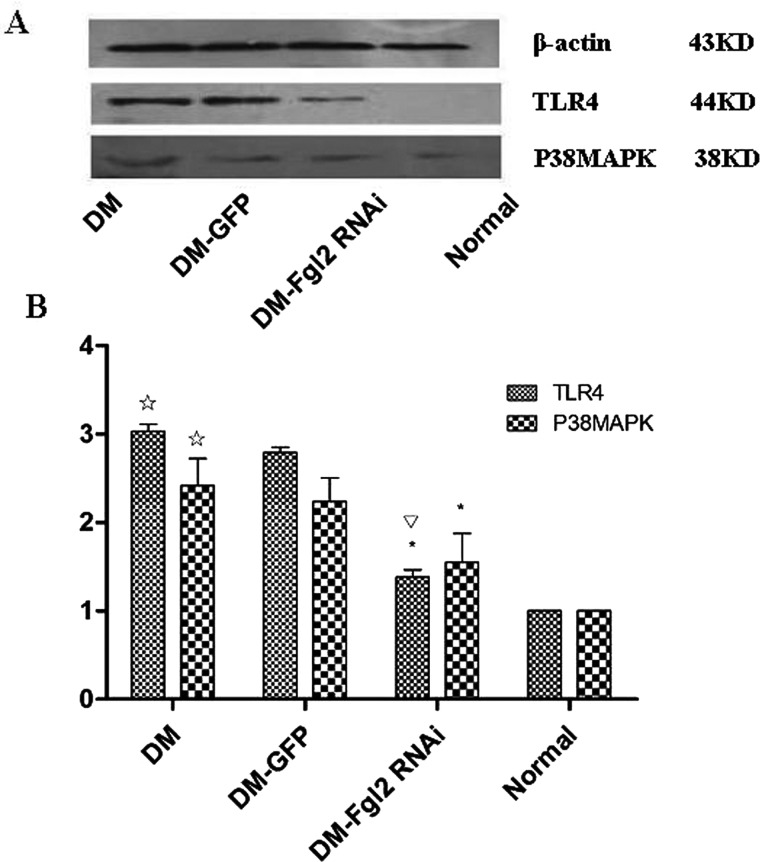
Expression of TLR4 protein and p38 MAPK proteins β-Actin is used as a standard control. The expression of TLR4 decreased significantly in DM–Fgl2–RNAi group compared with GFP group and DM group (*P*<0.05). The expression of p38 MAPK in DM–Fgl2–RNAi group decreased significantly compared with GFP group and DM group (*P*<0.05). Data are given as the means±S.D.s. ^#^*P*<0.05 compared with normal; *P<0.05 compared with DM–Fgl2–RNAi, ^☆^*P* > 0.05 compared with GFP group, ^▽^*P*<0.001 compared with normal; ^＊^*P* > 0.05 compared with normal.

## DISCUSSION

Our previous study found that Fgl2 gene silencing activates angiopoietin/Tie system and induces myocardial microvascular endothelial cell proliferation and cell migration [1], which indicates that Fgl2 is involved in angiogenesis. In addition, many studies found Fgl2 is involved in apoptosis, inflammatory response. As we know, diabetes is closely associated with apoptosis, angiogenesis and coagulation [8–10]. It is unclear what role Fgl2 plays during the process of DCM. In the present study, our data show that Fgl2 gene silencing improves the heart function of diabetic rats, but there has no improvement on blood glucose, which indicated that the effect of Fgl2 gene silencing on diabetic rats is not associated with glucose level. These results indicate that Fgl2 is a potent target for the treatment of DCM.

Previous studies have shown that diabetes mellitus alters cardiac structure and function independently of coronary artery disease and systemic hypertension, a condition known as DCM. The pathological mechanism of DCM is considered to involve myocardial apoptosis and necrosis [13,14]. In the present study, we found myocardial apoptosis and necrosis significantly increased in diabetic rats, this is consistent with previous studies [13,14]. We found that Fgl2 gene silencing inhibited myocardial apoptosis and necrosis, apoptosis was induced by diabetes mellitus via modulation of the bcl2 and bax; we first showed that Fgl2 gene silencing induced a decrease of bcl2 and bax, whereas bax expression decreased more than the bcl expression. The present study is the first study that focus on myocardial apoptosis induced by diabetes mellitus and showed that Fgl2 may be a potent target to treat diabetes mellitus. We did not know the cross-talk between Fgl2 and high glucose; in the present study, we found that Fgl2 silencing has no improvement on glucose. Further studies should be performed to show the relationship between Fgl2 and diabetes.

TNFα is a potent pro-inflammatory cytokine and an important mediator of inflammatory tissue damage. TNFα also has an immunoregulatory role [15]. Some study showed that TNF-α up-regulates Fgl2 expression in rat myocardial ischaemia/reperfusion injury [16]. In the present study, there was a significant increase in TNFα level in the diabetic rats compared with the control group, whereas Fgl2 gene silencing decreased the TNFα level. We showed the interrelation of TNFα and Fgl2 and the molecular mechanism which Fgl2 as a therapeutic target for the treatment of DCM.

Increasing studies in the recent years have provided strong evidence in supporting an essential role of TLR4 and p38 MAPK signalling in the pathogenesis of diabetes [17,18]. Animal studies have shown that TLR4 deficiency or loss-of-function mutation of TLR4 in mice conferred protection against lipid-induced insulin resistance [19]. *In vitro* studies have demonstrated that the activation of TLR4 led to increased expression of pro-inflammatory cytokines [20,21]. In the present study, we demonstrated that Fgl2 gene silencing down-regulated TLR4 expression and down-regulated p38 MAPK signalling, contributed to improve the function of the heart of diabetic rats.

In summary, we demonstrated that Fgl2 gene silencing inhibits apoptosis and improves heart function of STZ-induced diabetes rats, the possible mechanism maybe that Fgl2 gene silencing reduces the TNF-α levels, decreases the expression of bcl2, bax, TLR4 and p38 MAPK, but it has no effect on blood lipid. In conclusion, Fgl2 is a potent target to treat DCM. The preliminary data, although very encouraging, need to be well discussed and further study should surely be continued. It is possible that further studies will provide clues for better understanding of Fgl2 for prevention of DCM.

## Abbreviations

bax: bcl-2-associated X
bcl2: B-cell lymphoma-2
DCM: diabetic cardiomyopathy
EF: ejection fraction
Fgl2: fibrinogen-like protein 2
FS: fractional shortening
HE: hematoxylin and eosin
HR: heart rate
LSD: Fisher's Least Significant Difference
LV: left ventricular
MAPK: mitogen-activated protein kinase
STZ: streptozotocin
TLR4: toll-like receptors 4
TNF: tumour necrosis factor
TUNEL: terminal deoxynucleotidyl transfer-mediated dUTP nick end-labelling
